# Designing and development of multi-epitope chimeric vaccine against *Helicobacter pylori* by exploring its entire immunogenic epitopes: an immunoinformatic approach

**DOI:** 10.1186/s12859-023-05454-2

**Published:** 2023-09-22

**Authors:** Anand K. Keshri, Rimanpreet Kaur, Suraj S. Rawat, Naina Arora, Rajan K. Pandey, Bajarang V. Kumbhar, Amit Mishra, Shweta Tripathi, Amit Prasad

**Affiliations:** 1 School of Basic Sciences, Indian Institute of Technology Mandi, Himachal Pradesh, Mandi, 175005 India; 2 Department of Medical Biochemistry and Biophysics, Karolinska Institute, 17177 Stockholm, Sweden; 3 Department of Biological Sciences, NMIMS, Mumbai, 400056 India; 4 Cellular and Molecular Neurobiology Unit, Indian Institute of Technology Jodhpur, Jodhpur, Rajasthan 342011 India

**Keywords:** *Helicobacter pylori*, Vaccine, Multi-epitopes, Infection, Immunoinformatics

## Abstract

**Background:**

*Helicobacter pylori* is a prominent causative agent of gastric ulceration, gastric adenocarcinoma and gastric lymphoma and have been categorised as a group 1 carcinogen by WHO. The treatment of *H. pylori* with proton pump inhibitors and antibiotics is effective but also leads to increased antibiotic resistance, patient dissatisfaction, and chances of reinfection. Therefore, an effective vaccine remains the most suitable prophylactic option for mass administration against this infection.

**Results:**

We modelled a multi-chimera subunit vaccine candidate against *H. pylori* by screening its secretory/outer membrane proteins. We identified B-cell, MHC-II and IFN-γ-inducing epitopes within these proteins. The population coverage, antigenicity, physiochemical properties and secondary structure were evaluated using different *in-silico* tools, which showed it can be a good and effective vaccine candidate. The 3-D construct was predicted, refined, validated and docked with TLRs. Finally, we performed the molecular docking/simulation and immune simulation studies to validate the stability of interaction and in-silico cloned the epitope sequences into a pET28b(+) plasmid vector.

**Conclusion:**

The multiepitope-constructed vaccine contains T- cells, B-cells along with IFN-γ inducing epitopes that have the property to generate good cell-mediated immunity and humoral response. This vaccine can protect most of the world’s population. The docking study and immune simulation revealed a good binding with TLRs and cell-mediated and humoral immune responses, respectively. Overall, we attempted to design a multiepitope vaccine and expect this vaccine will show an encouraging result against *H. pylori* infection in in-vivo use.

**Supplementary Information:**

The online version contains supplementary material available at 10.1186/s12859-023-05454-2.

## Background

*Helicobacter pylori* is a gram-negative bacterium categorized as a group 1 carcinogen by WHO. It has evolved with a perfectly suited armamentarium to colonise in the highly acidic environment of the human gut [1]. It is mainly transmitted through direct contact with faecal matter, vomit, and saliva of the infected person, or contaminated water/food. Its colonization generally occurs in childhood, at the age ≤ 10 years, and once established, it can persist throughout the life [2]. The colonisation and infection of this bacteria are usually asymptomatic, but it may cause acute and chronic inflammation of gastric epithelia, which can eventually develop into severe forms of disease such as chronic gastritis, gastric ulceration, adenocarcinoma, and gastric lymphoma [2]. Gastric cancer is the 3rd deadliest and the 5th most frequently detected malignancy in the world, leading to > 19.1 million DALYs. It is estimated that > 50% of individuals are globally infected with this bacterium. The incidence of *H. pylori* in Western countries ranges from 30 to 50% but reaches an alarmingly very high level of 85–95% in developing countries. Southern Asian countries like India and Pakistan have the highest number of incidents (63.5% and 81%, respectively) [3].

The Toll-Like Receptors (TLRs) are the Pathogen Recognition Receptors (PRRs) that have been extensively studied and reported to identify many distinct evolutionary conserved molecular patterns of pathogens, known as Pathogen Associated Molecular Patterns (PAMPs). In *H. pylori* infection, TLRs have shown a significant role in sensing and recognising various antigens, initiating a cascade of signalling pathways and immune responses in host cells [4]. Additionally, the level of antimicrobial peptide, β-defensins, was observed to be elevated during *H. pylori* infection [5]. However, *H. pylori* infection is currently treated by a combination of proton pump inhibitors and antibiotics for at least a week, but administering high number of pills, especially antibiotics, had its consequences such as patient dissatisfaction and increased antibiotic resistance [6]. Increased antibiotic resistance among *H. pylori* patients is a major global concern and a big challenge for its global eradication initiative. Moreover, a prolonged antibiotic treatment regime can also potentially disrupt the gut microbiota symbiosis, leading to other gastric/physiological problems. Furthermore, antibiotics or drug administration cannot provide protection from reinfection. Therefore, an effective vaccine remains the most suitable prophylactic option for mass administration against *H. pylori*. Several research groups across the globe had come out with various kind of vaccines against this bacterium, but none is available under licensed production, and only one in phase III clinical trial [7, 8]. The recent advancements in the immunoinformatic field provide an array of tools for the efficient and effective designing and development of novel potential vaccine candidates in a cost-effective manner. The immunoinformatic tools are reasonably effective, rapid, and accurate in identifying epitopes and developing a stable and accurate multi-epitope subunit vaccine against pathogens [9].

In our work, we intended to create a multi-epitope chimeric vaccine against *H. pylori* that will help in reducing the *H. pylori* infection and thus associated morbidity/mortality. Here we used the bacterial secretion pathway to identify the possible excretory and membrane proteins. We especially used these secretory/outer membrane proteins to find the potential candidate for the T and B-cell epitopes. The constructed vaccine candidate was then evaluated for their allergenicity, physicochemical characteristics, and structure validation. We further used molecular docking for confirming the final vaccine interaction potential with TLRs. The candidate vaccine was finally cloned in plasmid for expression by *in-silico* cloning.

### Protein collection and ESP identification

**Fig. 1:**
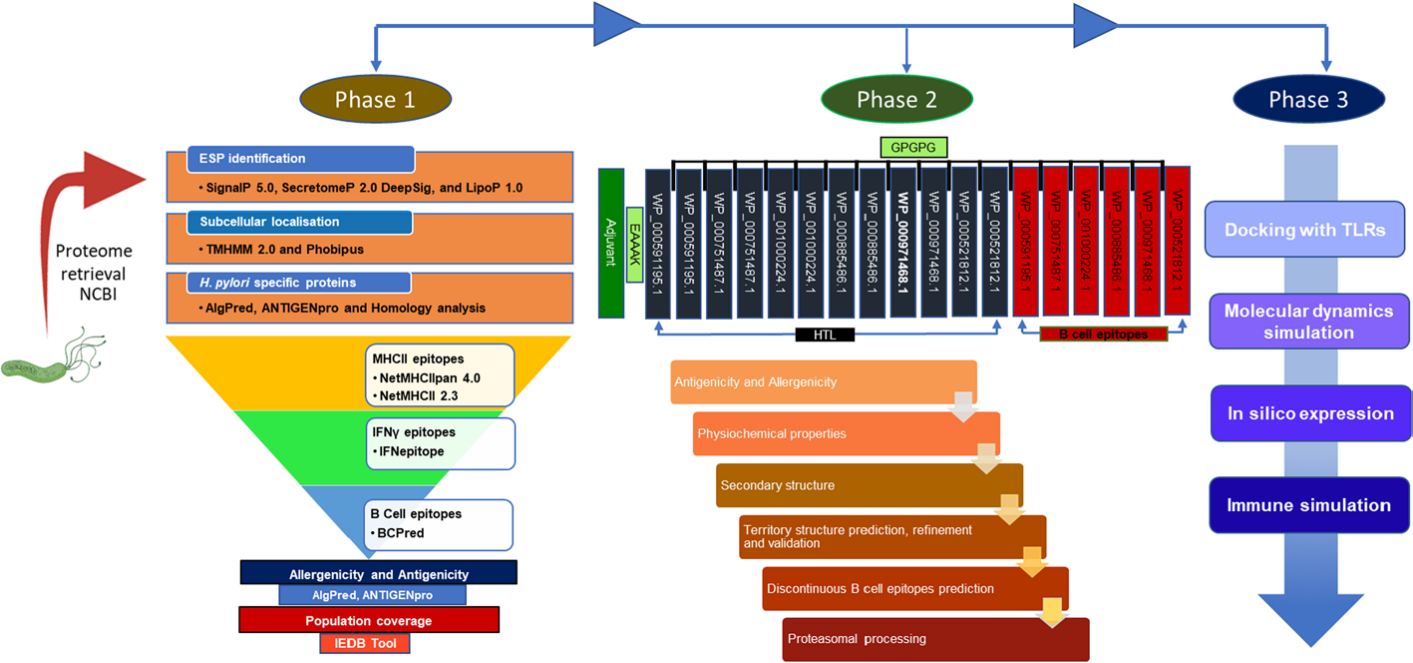
Phase wise schematic presentation of the detailed workflow used in this study. Phase 1 consisted of the selection of proteins and epitopes. Phase 2 involved the construction of a vaccine candidate, structure prediction, and validation. Phase 3 involved docking with the immune receptor, immune simulation, and *in-silico* expression

The pipeline used for the construction of vaccine candidate is provided in Fig. 1. We retrieved the *H. pylori* proteome obtained from the NCBI, which contains 1465 protein sequences. Out of these 1456 sequences, 228 non-classical proteins were selected by SecretomeP-2.0 server, having a SecP score greater than 0.5. Out of 228 proteins, 88 were identified as having SP (Sec/SPI) signal peptides. The DeepSig server was used to discriminate these 88 proteins from those with N-terminal transmembrane segments and others by their signal peptides. To further eliminate any errors, the LipoP 1.0, TMHMM 2.0, and Phobius software were employed, resulting in the identification of 70 proteins that lacked cytoplasmic and transmembrane regions.

### Identifying *H. pylori*-specific proteins

All 70 selected proteins exhibited good antigenic scores and negative allergen scores. From this pool of proteins, 25 were chosen based on their less than 50% identity to humans and high antigenic scores (Additional file 1: Table S1).

### Prediction of helper T-cell, B-cell and IFN-γ inducing epitopes

From the selected 25 protein candidates, a total of 9601 and 11,090 HTL epitopes were predicted using the NetMHCIIpan-4.0 and NetMHCII 2.3 servers, respectively. We chose 130 epitopes having common sequences from both servers with ≤ 3 percentile ranks. Among these 130 common epitopes, only 25 epitopes were found to share common amino acids for IFN-γ-inducing epitopes. Additionally, we identified 21 B-cell epitopes from the chosen proteins with an epitope score > 0.95. In the final selected epitopes list (Tables 1 and 2), we included antigenic epitopes while discarding allergenic epitopes (Tables 1 and 2).

**Table 1:** Comprehensive list of* H. pylori* proteins selected for the design of a potential vaccine each protein is accompanied by its respective accession ID and HTL cell epitopes

*H. pylori protein*	Accession Id	MHCII epitopes	Alleles	% Binding	IFN-y	Antigenic score
Hop family outer membrane protein HopL	WP_000591195.1	KHESLRAYENAKDYD	HLA-DQA10102-DQB10401	0.27	Positive	0.6429
			HLA-DQA10103-DQB10202	1.1		
			HLA-DPA10201-DPB10901	1.23		
		GEIYNMYAHTAAHKT	HLA-DQA10102-DQB10401	0.17	Positive	0.7245
			HLA-DQA10103-DQB10202	1.46		
			HLA-DQA10103-DQB10202	2.45		
Hop family adhesin HopQ	WP_000751487.1	ISNVYSAKVNTANFQ	HLA-DQA10102-DQB10401	0.16	Positive	1.2648
			HLA-DQA10103-DQB10202	1.39		
		ARPKKKDSHHAAQHG	HLA-DPA10201-DPB10901	1.59	Positive	1.1025
			HLA-DQA10103-DQB10202	2.46		
			HLA-DQA10102-DQB10401	2.58		
Flagellar hook protein FlgE	WP_001000224.1	NEPIDAITNRKLNIS	HLA-DQA10103-DQB10202	1.27	Positive	0.4278
			HLA-DQA10102-DQB10401	1.47		
		SNNIANVNTLGYRSN	HLA-DQA10103-DQB10202	1.13	Positive	0.7005
			HLA-DQA10102-DQB10401	1.8		
Flagellin A	WP_000885486.1	NSNQTGVRAHASVIT	HLA-DPA10201-DPB10901	1.17	Positive	1.3663
			HLA-DQA10102-DQB10401	0.93		
		SGANYNAVIASGNQN	HLA-DQA10102-DQB10401	0.51	Positive	0.7481
			HLA-DQA10103-DQB10202	1.95		
			HLA-DQA10102-DQB10401	1.4		
Outer membrane protein	WP_000971468.1	HTNFSNSRAANAISP	HLA-DQA10102-DQB10401	0.91	Positive	0.6775
			HLA-DQA10103-DQB10202	2.51		
		ATAGFFVGVNFAGNT	HLA-DPA10105-DPB10801	1.53	Positive	0.4978
			HLA-DPA10105-DPB10801	0.78		
Septal ring lytic transglycosylase RlpA family protein	WP_000521812.1	QAYLRSAGADVSYRR	HLA-DQA10102-DQB10401	1.51	Positive	0.7258
			HLA-DQA10102-DQB10401	2.65		
		ARNQVQNAQNQANNY	HLA-DQA10102-DQB10401	2.121096	Positive	0.4865
			HLA-DQA10102-DQB10401	0.118137		

The table also provides details such as HLA class II alleles associated with selected epitopes along with their binding percentage. Further, it includes IFN-γ inducing positivity and the antigenic score of each epitope

**Table 2:** Comprehensive list of* H. pylori* proteins selected for the design of a potential vaccine. Each protein is accompanied by its respective accession ID

*H. pylori* protein	Accession Id	B-cell epitopes	Score	Antigenic score
Hop family outer membrane protein HopL	WP_000591195.1	TSNTNSANNTNS	1	1.5261
Hop family adhesin HopQ	WP_000751487.1	TTDGGKNSCQTF	0.995	1.2165
Flagellar hook protein FlgE	WP_001000224.1	NDPTSSPTSKRK	0.984	0.9571
Flagellin A	WP_000885486.1	DVTGNFNANVKS	0.988	1.0303
Outer membrane protein	WP_000971468.1	GTITCGDTTPAS	0.999	0.9862
Septal ring lytic transglycosylase RlpA family protein	WP_000521812.1	KGYNHSQEVEKV	0.997	0.8083

The table also provides details such as B-cell epitopes derived from selected proteins and for each B-cell epitope, the epitope's score and antigenic score were given

Based on the above criteria, we selected the following proteins for vaccine: WP_000591195.1 (Hop family outer membrane protein HopL), WP_000751487.1 (Hop family adhesin HopQ), WP_001000224.1 (flagellar hook protein FlgE), WP_000885486.1 (flagellin A), WP_000971468.1 (outer membrane protein), and WP_000521812.1 (septal ring lytic transglycosylase RlpA family protein).

### Population coverage and toxicity prediction of selected vaccine candidate

**Fig. 2:**
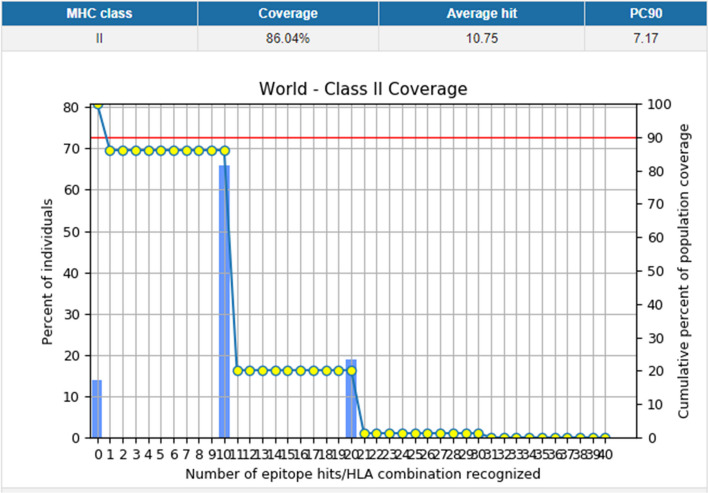
The population coverage analysis of selected MHC II epitopes based on HLA interactions worldwide shows that these epitopes provide coverage for 86.04% of the global population, with an average hit of 10.75. The bar represents the population coverage for each epitope, while the cumulative percentage of population coverage is represented by a line graph (−o–)

Considering allelic variation among world population is crucial for a globally effective vaccine. We utilized the world population coverage tool provided by the IEDB server, which showed that our vaccine candidate provided coverage for 86.04% of the global population, with an average effectiveness rate of 10.75 (Fig. 2). Additionally, the final selected epitopes underwent toxicity assessment, and none were found to possess toxic properties. Thus, these epitopes were deemed safe for inclusion as vaccine candidates (Additional file 1: Table S3).

### Merging epitopes for multiepitope vaccine

To create a multi-epitope vaccine, we combined the 12 overlapping sequences of T-cell epitopes with IFN-γ epitopes and six B-cell epitopes using the linker GPGPG. The adjuvant β-defensin (PDB ID: 1FD3_A) was obtained from the PDB database and linked epitopes were strategically placed at the C-terminal of the adjuvant using the EAAAK linker to amplify the immune response. The final composition of the vaccine involved a sequence of 405 amino acid residues.

### Physiochemical properties estimation and secondary (2D) structure prediction

**Fig. 3:**
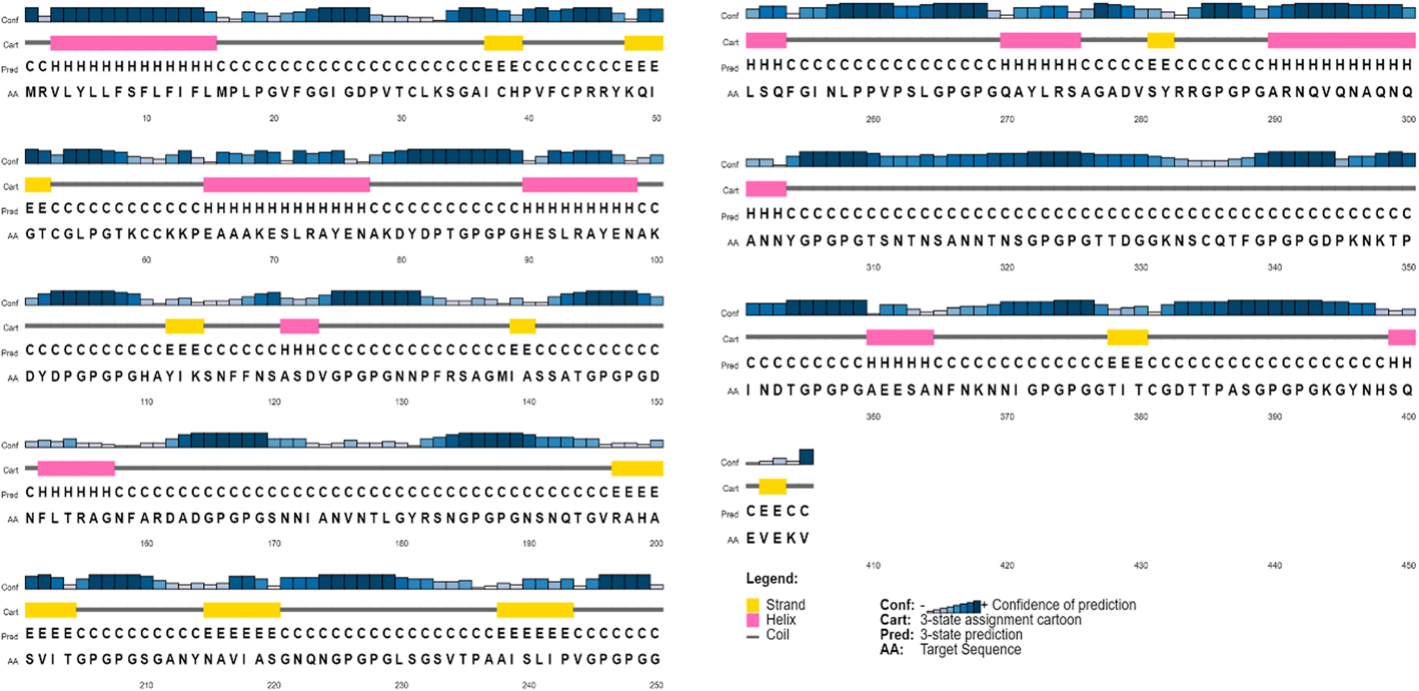
The graphical representation of the predicted secondary structure elements from the PSIPRED view tool, including α-helix, β-sheet, and coils, with percentages of 13.58%, 11.85%, and 74.57%, respectively

The vaccine candidate was found to be devoid of any allergic reactions. The constructed vaccine had a MW (molecular weight) and pI (isoelectric point) of 40.5 kDa and 9.38, indicating its basic nature. Furthermore, the calculated instability index was 24.23, signifying the stability of this protein, along with a GRAVY (grand average of hydropathy) score of − 0.589 and an aliphatic index of 54.16. The determined ½ life of the developed vaccine was determined to be more than 30 h in both* E. coli* and mammalian cells, while it was 20 h for *Saccharomyces cervisiae*. These properties confirmed the stability of the designed vaccine candidate. The 2D structure of the putative candidate vaccine consisted of α-helix, β-sheet, and coil with percentages of 13.58%, 11.85%, and 74.57%, respectively (Fig. 3).

### Prediction, refinement and authentication of tertiary (3D) structure

**Fig. 4:**
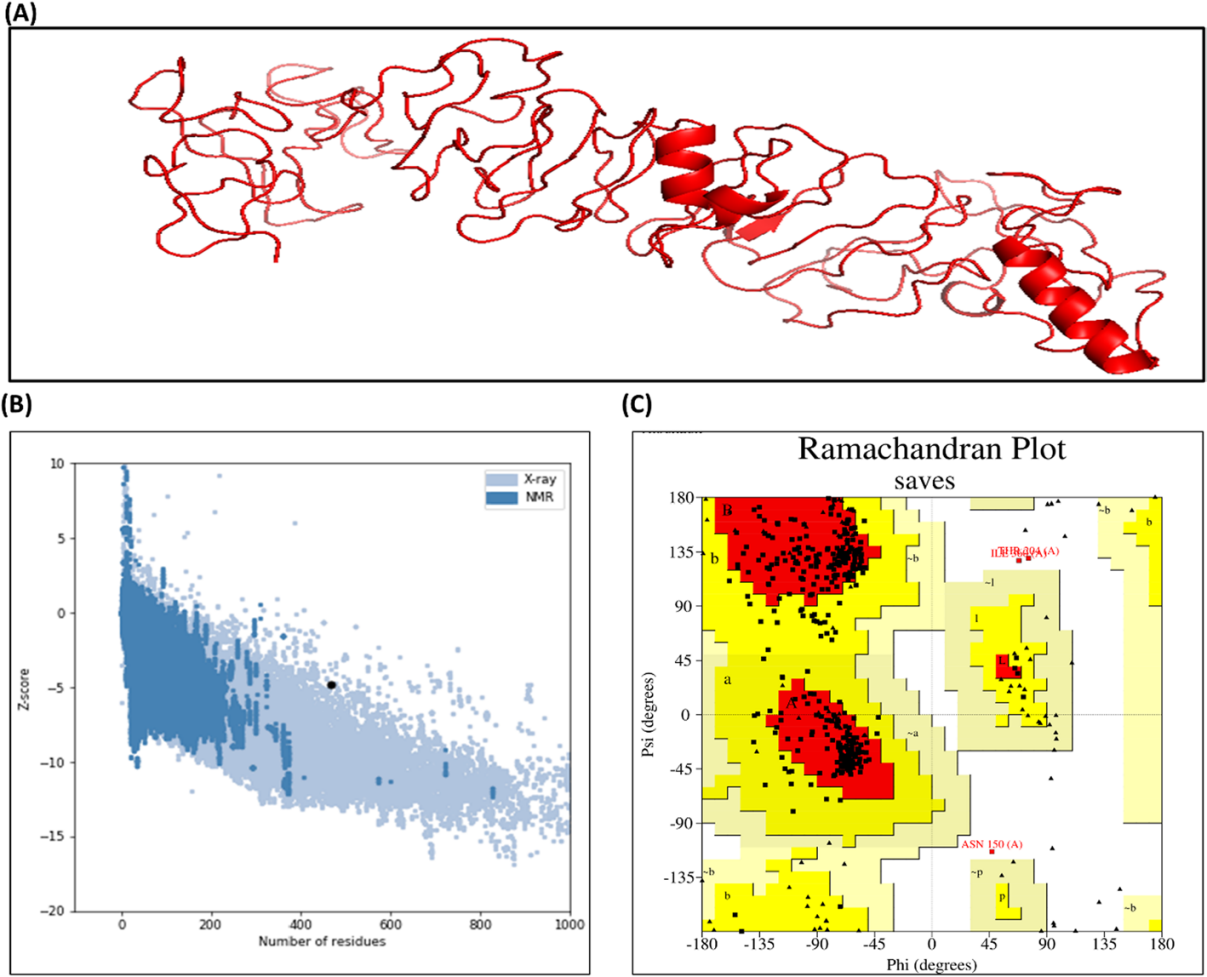
The final structure of the constructed vaccine and its validation. **A** The refined 3D model of the multi-epitope vaccine obtained from RaptorX shows the spatial arrangement of the epitopes within the vaccine structure. **B** ProSA structure validation displays a − 4.8 Z score, indicating the overall quality of the construction which is within an acceptable range. **C** Ramachandran plot analysis reveals that 82.4% of the amino acid residues in the vaccine structure are located in the favoured region, 16.6% of the residues fall within the allowed region, while only 1% are found in the disallowed region, indicating energetically favourable conformation, acceptable conformations, potentially unfavourable conformations, respectively

RaptorX was employed for the construction of the 3D model of the vaccine (Fig. 4A). Out of the five models generated, the first model with an RMSD value of 9.8884 was chosen for refinement using the Galaxy Refine software, which had the lowest Z-score value of − 4.54 determined by ProSa-web tool (Fig. 4B). The refined model had an RMSD value of 0.259, clash score of 17.1, MolProbity score of 2.178, and a low percentage of poor rotamers (0.3%). Furthermore, 93.7% of the amino acid (AA) residues in the model were in the Ramachandran favoured region. The total potential energy of the construct was reduced using the YASARA tool, resulting in a more negative Z-score of − 4.8 and improved overall protein quality, as assessed on the Prosab-web server (Additional file 1: Fig. S1A). Ramachandran plot confirmed that 82.4% of the AA residues were present in the favoured region, with 16.6% and 1% of residues in the disallowed region, respectively (Fig. 4C). Additionally, the 3D-1D amino acid score was 0.2, and the overall ERAAT factor was 77.455 (Additional file 1: Fig. S1B and C).

### Discontinuous B-cell epitope prediction

**Fig. 5:**
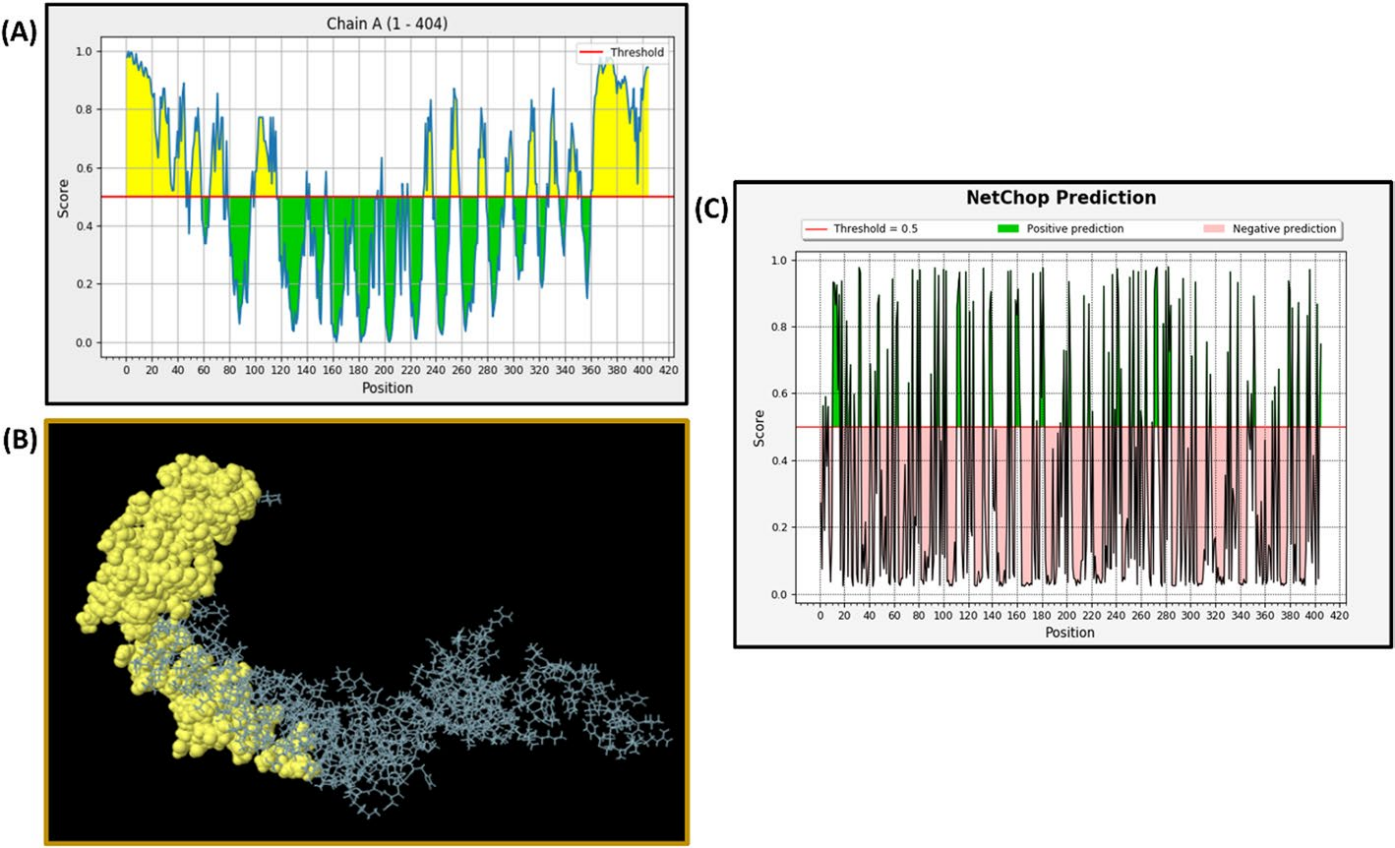
**A** The predicted discontinuous B-cell epitopes from the ElliPro server are shown here. The epitopes are depicted as a graph, where the X-axis represents the amino acid position, and the Y-axis represents the score. The yellow region indicates potential B-cell epitopes with scores exceeding the threshold of 0.5. **B** A 3D representation of the discontinuous B-cell epitopes, where the residues are highlighted in yellow. **C** The proteasomal cleavage sites in the constructed vaccine, as predicted by the IEDB server. The green portion represents positive predictions with scores higher than the threshold of 0.5

The discontinuous B-cell epitopes were distributed all over the structure, with 180 residues distributed across 12 confirmations, scoring between 0.831 to 0.514. The size distribution of the epitopes varied from 7 to 84 amino acid residues (Fig. 5A and B).

### Proteasomal processing

It is crucial for our constructed vaccine to produce peptides that can bind to MHC I and activate cytotoxic T-cells. The proteasome cleavage analysis was conducted by the Proteasome Cleavage Prediction Server, and we identified a total of 93 proteasomal and 127 immunoproteasomal cleavage sites. Additionally, the Proteasomal Cleavage Prediction tool of the IEDB server predicted a total of 195 cleavage points. These findings suggest that our constructed vaccine has the potential to stimulate cytotoxic T-cells (Fig. 5C).

### Molecular docking

**Fig. 6:**
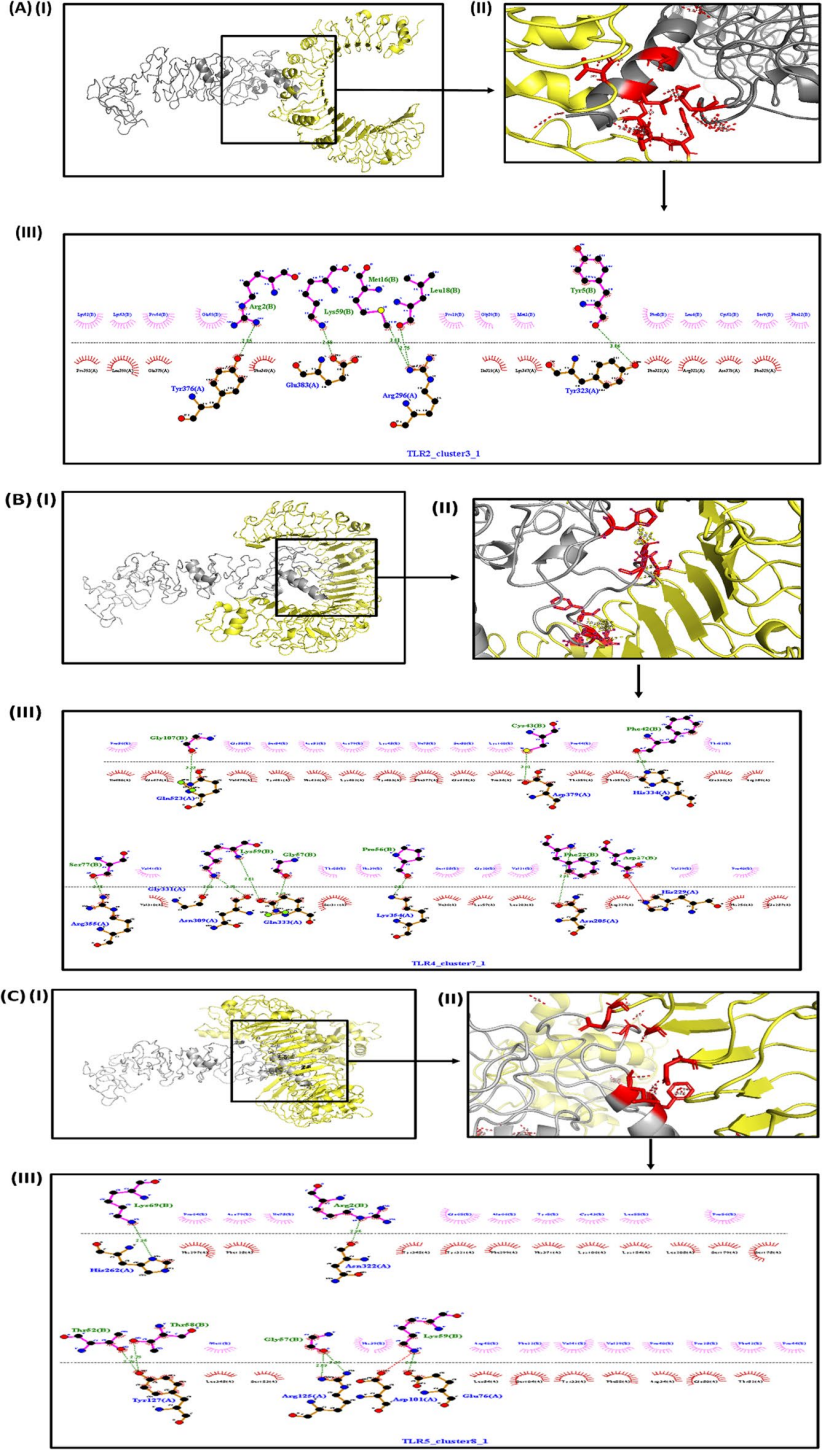
**A** The docked complex of TLR2 with the vaccine construct exhibits a binding energy of − 23.45 kcal/mol indicating the strength of interaction. (I, II) The structure of the complex is visualised by Pymol software, which enables a view of the spatial arrangement and and interaction of the TLR2 receptor (Chain A) and the vaccine construct (Chain B) within the complex. (III) The interaction between the two molecules is further examined using LigPlot + , which provides an insightful visualization of the specific interactions and binding interfaces between TLR2 and the vaccine. **B** The docked complex of TLR4 with the vaccine construct shows a binding energy of − 25.87 kcal/mol. (I, II) The structure is visualized using Pymol software. (III) The interaction is visualized using LigPlot + (Chain A—TLR and Chain B—Vaccine construct). **C** A docked complex of TLR5 with the vaccine construct demonstrates a binding energy of − 33.24 kcal/mol. (I, II) The structure is visualized using Pymol software. (III) The interaction is visualized using LigPlot + (Chain A—TLR and Chain B—Vaccine construct)

TLRs play a significant role as molecular receptors in inducing immunity against pathogens, and TLRs 2, 4, and 5 have been associated with *H. pylori* infection. Therefore, we selected these TLRs to investigate the binding of the constructed vaccine as this is essential for induction of immune response. The ClusPro server provided several docked models, and we chose the best model with the lowest energy. From this docked complex, we identified the interactive residues and utilized them in the Haddock server. The Haddock server yielded 8 clusters, from which we selected the best-docked complex based on the lowest score (Additional file 1: Table S2) among all clusters. The Pymol assisted in visualizing the interacting partners, and the LigPlot + server aided in identifying hydrogen and other interactions. The interactions between TLRs and the vaccine were summarized in Fig. 6A–C, and the interaction scores of different clusters were described in Additional file 1: Fig. S2A–C.

### MD simulation

**Fig. 7:**
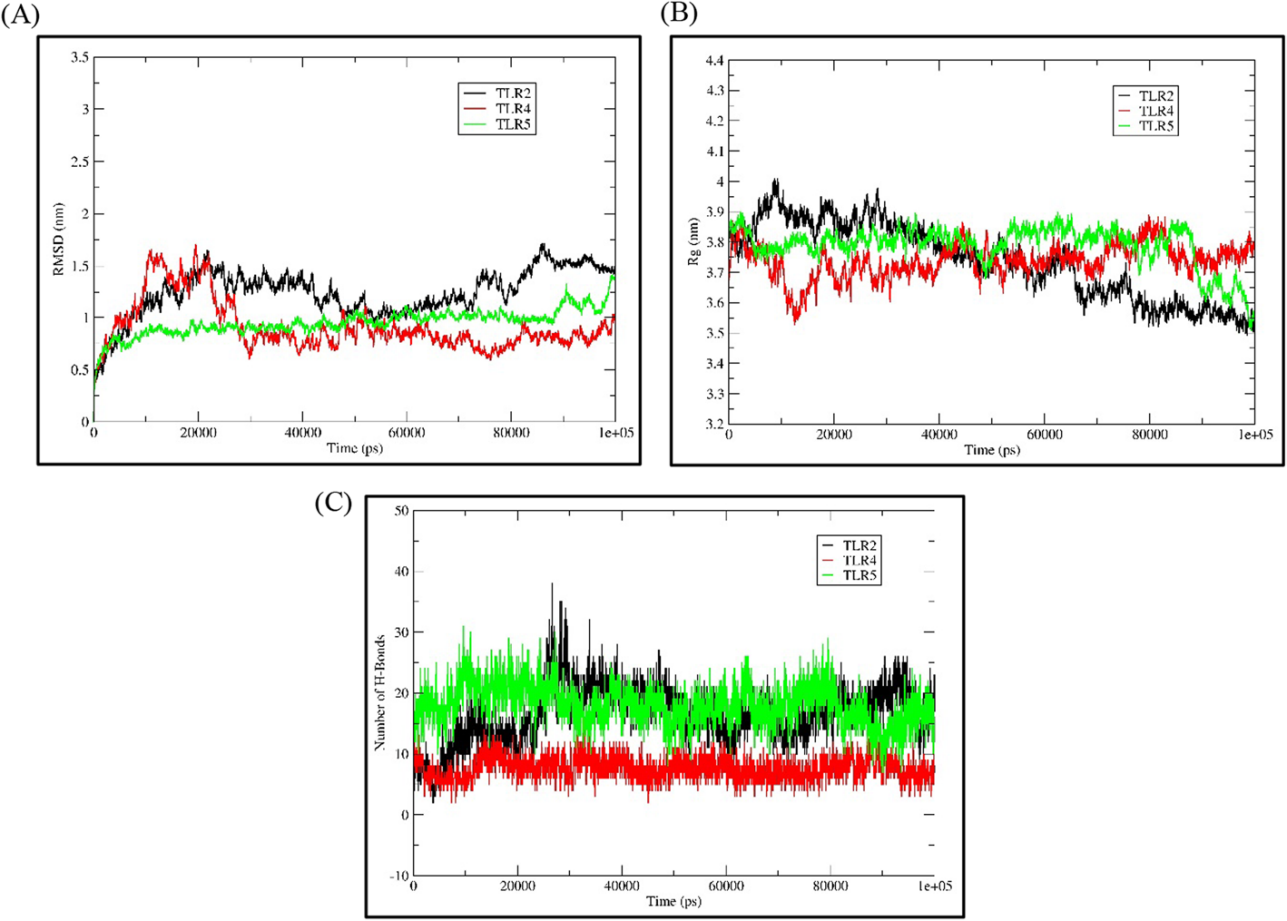
**A** The RMSD plot demonstrates the stability of the vaccine-receptor complexes, including -TLR2 (Vaccine TLR-2 complex), TLR4 (Vaccine TLR-4 complex), and TLR5 (Vaccine TLR-5 complex). The plot shows the fluctuations in the RMSD values over time, indicating the stability of the complexes during the simulation. **B** The Rg plot illustrates the compactness of the complexes, representing the average distance of the vaccine-receptor complex components from their centre of mass. **C** The Number of H bond plot depicts the number of hydrogen bonds formed between the vaccine and receptor over the course of the simulation

To evaluate the stability of the complexes formed between the vaccine and TLR2, TLR4, and TLR5, we performed MD simulations for 100 ns using GROMACS. We analysed the Root Mean Square Deviation (RMSD), radius of gyration (Rg), number of hydrogen bonds (H-bonds), and for the docked complexes. The RMSD plot reflected the stability of the structures (Fig. 7A–C). The RMSD values for the TLR2-vacc, TLR4-vacc, and TLR5-vacc complexes were 1 ± 0.5 nm. The TLR5-vacc complex stayed stable throughout the 100 ns simulation, while the TLR4-vacc complex showed some variation until 30 ns and then attained stability. The TLR2-vacc complex also exhibited some variation, although TLR4-vacc and TLR2-vacc complexes showed good stability. The Rg values indicate the compactness of the structures, with the TLR5-vacc complex showing an Rg value of approximately 3.8 ± 0.05 until 90 ns, and the compactness increasing thereafter (Fig. 7A). The TLR2-vacc and TLR4-vacc complexes showed few fluctuations, but overall, they demonstrated good compactness (Fig. 7B). All three docked complexes exhibited good interactions, as reflected by the H-bonds, further supporting the compactness of the structures (Fig. 7C). Taken together, the vaccine-receptor complexes exhibited good stability and interactions, suggesting that the vaccine has the ability to attach to the receptors with strong interactions and generate a significant immune response.

### Immune simulation

**Fig. 8:**
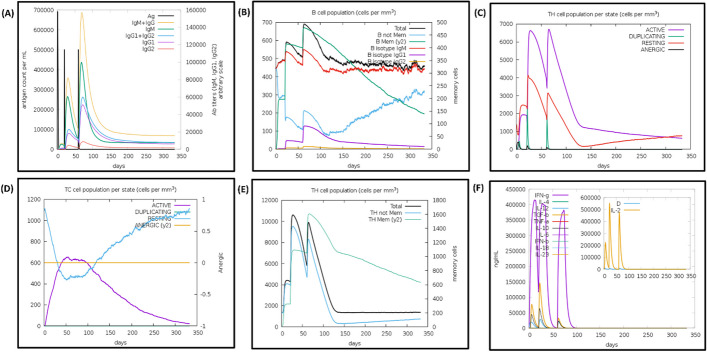
C-ImmSim presentation of an in silico immune simulation with the constructed vaccine. **A** Increased immunoglobulin production in response to the vaccine injection, indicating an immune response. **B** The population dynamics of B cells following the three vaccine injections, provide insights into the immune response generated by the vaccine. **C** The evolution of the TH cell population, with each vaccine injection, over the course of the immune simulation. However, the population decreases when the concentration of antigen decreases. **D** The populations of T-cytotoxic cells in different states following the vaccine injections, indicating their involvement in the immune response elicited by the vaccine. **E** The TH memory cell population, shows its changes over time. The memory cell population increases specifically after the third injection, while the total cell population continues to increase with each injection. **F** The levels of cytokines, measured by the Simpson index, following the three vaccine injections

The immune stimulation resulted in an increased secondary and tertiary immune response during in silico injection simulation. We observed elevated titers of IgM, IgM + IgG, IgG + IgG, and IgG1 antibodies (Fig. 8). Additionally, we observed an increased level of the B-cell population, particularly the memory B-cell population. Similar trends were observed for T-cytotoxic and helper cells too. Importantly, no allergic response was found, while parameters related to the innate immune cell response, such as the levels of IFN-γ, IL-2, and TGF-β, were elevated. These responses suggest that the constructed vaccine has the potential to mount a robust immune response with a long-lasting humoral response, and it will not cause any allergic reaction (Additional file 1: Fig. S3).

### In-silico cloning

The Jcat server optimised the sequence in respect of *E. coli* for maximum expression. The optimised sequence was 1215 nucleotides long with a Codon Adaptation Index (CAI) of 1, and 57.11% of GC content which was in the range of optimal range (30–70%) inside the *E. coli* host. Finally, we designed a pET28b (+) recombinant plasmid by inserting the adapted sequence by using Snap Gene software between *NdeI* and *BmtI* restriction site (Additional file 1: Fig. S4).

## Discussion

At present, vaccines are the most effective weapon to fight against infectious diseases and had helped to save millions of lives so far. The conventional approach to vaccine development is time-consuming and expensive. In contrast, the immunoinformatics approach is comparatively inexpensive, more effective, and less time-consuming [10–12]. This approach has been employed to design vaccines against bacterial, viral, and parasitic pathogens [10, 13, 14]. The computational approach helps in identifying new immune-protective antigens and immunodominant T and B-cell epitopes, that act as a key factor in the initiation of both arms of the host immune system (cellular and humoral immunity), with a high degree of certainty and it makes them more effective [15].

As of now, it’s clear that the secretory proteins released by pathogens plays an important role in defining the outcome of an infection [13, 16]. This approach will rule out missing any protein with good antigenic potential, thereby increasing the chance of success. Hence, we looked to the whole proteome of the *H. pylori* 135 Puno strain to identify secretory/outer membrane proteins. We identified 70 secretory proteins, by classifying them into classical and non-classical secretory proteins, lipoprotein, and transmembrane regions using different online servers.

The two well-studied secretory proteins of *H. pylori*, VacA and CagA, are considered major virulence factors. These two virulent proteins are known to affect multiple host signalling pathways and can also modulate the immune response and were present in our selection [17]. The identified 70 secretory/outer membrane proteins did not show any transmembrane domain which confirmed their secretory/outer membrane nature. Then we screened 25 unique, non-allergic and antigenic proteins of *H. pylori* which have less than 50% sequence homology with the host proteome to minimize the cross immune-reactivity of the resultant vaccine.

Next, we collected the HTL MHCII epitopes against human alleles HLA-DQA1*01*02, HLA-DQA1*02*01, DQB1*01*401, HLA-DQA1*01*03, HLA-DPA1*01*01 and HLA-DPB1*09*01. The expressions of these alleles have been shown in *H. pylori*-related gastric cancer, gastric ulcer, gastritis etc. [18–20]. The selection of epitopes with high affinity to these alleles ensured the efficient epitope presentation to T-cell receptors. The role of IFN-γ in *H. pylori* infection is still debatable, however, several reports are in favour of IFN-γ protecting the host against the infection [21]. Therefore, to improve our epitope selection we identified IFN-γ inducing epitopes within the selected MHCII epitopes and included them in the candidate vaccine (Table 1). Since B-cell has a crucial role in memory generation, we also screened the B-cell linear epitopes present in the selected proteins. To be a good epitope for vaccine, we selected those epitopes which are antigenic but not allergenic in nature (Table 2). Considering the population coverage, our selected epitopes should protect 86.04% of the global population, with an average effectiveness rate of 10.75 (Fig. 2). The finally selected protein antigens are HopL, HopQ, FlgE, flagellin A, outer membrane protein, and RlpA family protein. Some studies had shown the involvement of these proteins in disease pathogenesis such as HopL variant *H. pylori* were found to be intricately connected to the proliferation of GC [22], while the HopQ protein has been found more often in cag + strain and increases the adhesion of *H. pylori* to the AGS cells, leading to peptic ulcer conditions [23]. The FlgE, flagellin A and Omp are mainly involved in motility and colonisation [24]. Additionally, the participation of these proteins is important for the pathogenesis of *H. pylori,* and the vaccine construction using these proteins can be a new approach to help in the clearance and limit the colonisation of this bacterium.

The epitopes are small peptides containing 12–15 AAs only, these small peptides are considered less stable and are easy target for host proteases. Hence, injecting these individual peptides will not be a good strategy to initiate the immune response. So, we chose the GPGPG linker to combine the T and B-cell epitopes to make them of high molecular weight. The GPGPG linker has been widely used to combine recombinant vaccines against a range of pathogens, aiming to improve immunogenicity and efficacy of HTL epitopes [13, 25, 26]. The GPGPG linker, being a flexible linker, provides flexibility and independent movement of connected epitopes, maintaining conformational integrity by preventing misfolding and aggregation, and preventing stearic hindrance [26, 28, 29]. Conformational integrity is particularly important for vaccine antigens as it ensures antigenicity and native conformation during storage, transportation, and administration [27]. The GPGPG linker is utilized to combine T and B-cell epitopes, as these linkers allow the adjacent domains to be approachable while enhancing the solubility of the peptide. They also improve epitope separation and presentation while effectively preventing junctional epitope formation [25]. The β-Defensins have diverse roles, including acting as danger signals to create a pro-inflammatory response and promoting the maturation of antigen-presenting cells (APCs) [30]. It is naturally present in mucosal surfaces such as respiratory and gastrointestinal tracts and incorporating β-defensin as an adjuvant can also aid in mucosal delivery and strengthen the immune response at respiratory and gastrointestinal mucosal surfaces. It increases the acquired immune response by chemically absorbing the activity of immune cells and enhances the chemotactic activity of immature dendritic cells, T cells and other immune cells mobilisation and facilitates their maturation by increasing the antigen uptake. These properties can serve as a potent immunostimulant when used as an adjuvant along with vaccine administration [31]. Earlier, Ma XT et al. had demonstrated that a vaccine incorporating β-defensin 2 elicits potent antileukemia responses by activating innate and adaptive immune responses [32]. Additionally, it also promotes the infiltration of macrophages, NK cells, CD4+ T cells, and CD8+ T cells, and inhibits the growth of implanted murine melanoma in vivo [30]. The β-defensin 2 has also been shown to enhance the immunogenicity of vaccines against the influenza virus in mice [33, 34]. Studies have found high levels of β-defensin 2 in *H. pylori* infections [35–37]. To further improve the immunogenicity of peptides, β-defensin 2 was added at the peptide's N-terminal with an EAAAK linker. The EAAAK linker is a rigid peptide that efficiently separates the adjuvant from the functional domains, minimizing interference between epitopes and maintaining the functional properties of individual epitopes [27, 38].

The constructed vaccine has a MW of 40.4 kDa with a theoretical pI of 8.83, thereby denoting its basic nature. The computed instability index of 23.02 suggests its stability. Furthermore, we generated the secondary and tertiary structure by using PSIPRED and Raptor X software. We then refined the tertiary structure by using galaxy refine and YASARA, resulting in an improved overall quality of protein. The Ramachandran plot supports the tertiary model, with 82.4% AA in the favoured region, 16.6% in allowed and only 1% in the prohibited region. Furthermore, ERAAT (77.455) and 3D-1D (0.2) scores endorsed the overall quality of the tertiary model. The Ramachandran plot, ERAAT and 3D-1D scores have been widely utilised for the vaccine design, and a good score shows the promising quality of the predicted tertiary structure [26, 29].

Proteasomal cleavage of pathogenic proteins generates MHC I binding peptides, which are known to generate a cellular immune response against the parasites/pathogens. The multiepitope vaccine, with immunoproteasome cleavage sites, further signifies the relevance of the multiepitope vaccine (Fig. 5C). Additionally, we found some of the MHC II epitopes had shared sequences with MHC I epitope peptides. The Ellipro (IEDB server) predicted more cleavage sites (195) as compared to the proteasome option provided by Proteasome Cleavage Prediction Server (93). The potential disjointed B-cell epitope peptides were algorithmically calculated by using the ElliPro server, and we found discontinuous B-cell epitopes that maintain immunogenicity in the 3-D modelled multi-chimeric vaccine. Discontinuous epitopes are found on proteins with complex folded structures, and they often exhibit conformational specificity. Incorporating discontinuous epitopes in vaccine design can enhance the immune response and broaden the antibody repertoire. In vaccine formulation, assimilating both the linear and discontinuous epitopes increases the likelihood of generating antibodies that recognize the intact pathogen and its different conformations and can improve the efficacy of the vaccine [39].

TLRs play a crucial role in pathogen recognition and in initiating a cascade of signalling pathways for immune activation. Immune cells like epithelial, fibroblast and APCs (macrophages and dendritic cells), express TLRs involved in PAMPS recognition. Previous studies have shown the involvement of several TLRs in *H. pylori* infection, especially TLR2, TLR4 and TLR5 [40, 41]. The TLR5 sense the flagellin proteins, while TLR4 promotes cell surface binding of *H. pylori*. A diverse range of microbial components is recognised by TLR2 by virtue of its ability to form a heterodimer with TLR1, 6 and 10 [42, 43]. Hence, we selected these for docking studies (Fig. 6A–C and Additional file 1: Fig. S2A–C). Our constructed vaccine showed interaction with all three TLRs with several hydrogen and hydrophobic interactions with good binding energy. The MD simulation supports the structure which possesses good stability, compactness, and interactions, suggesting the vaccine can induce a good immune response upon binding to these receptors (Fig. 7).

Immunoreactivity and serological analysis were the first steps to validate the vaccine candidate, so immune stimulation experiments were carried out. Immune simulation results revealed an increased level of IgG1, IgG2 and IgM response after repeated exposure to antigens (Fig. 8), which was consistent with the previously published studies [44]. The memory B-cell and T-cell populations were also observed. Interestingly, our construct was found to induce the IL-2 and IFN-γ levels after the 1^st^ injection, and the expression of these cytokines remained at peak on subsequent injections. This indicates a good humoral response by maintaining a high number of TH cells and Ig production [45]. Cheng et al. demonstrated that the predicted result from the C-ImmSimm server aligns well with real-world experiments and can give a promising and consistent result [46]. They obtained a high level of IFN-γ+ T lymphocytes and IgG/IgG2a antibodies from the immunised mice, which were predicted by the C-ImmSimm server [46]. This supports our result, as the C-immSimm server also predicted the high level of IFN-γ and IgGs response from our constructed vaccine. Previous attempts have been made to develop vaccines using recombinant proteins, with some of them in the advanced stage, but no vaccine is yet commercially available for use [7, 8, 47]. Other researchers had also attempted to develop multi-epitope vaccines using immunoinformatic approaches by targeting several antigens of a bacterium e.g., outer membrane proteins such as HpaA, FlaA, FlaB, and Omp18 have also been used to make a vaccine candidate, along with 50S ribosomal proteins as an adjuvant, which have shown that the vaccine candidate interacted with TLR5 [48]. The combination of UreB, HpaA, and NapA protein along with melittin was also been used to design a vaccine [49]. Similarly, CagA, OipA, GroEL and VacA antigens were also used for vaccine construction, with β-defensin4A as an adjuvant [50]. In an another approach, to increase the potency of the vaccine the epitopes of UreB, VacA, CagA, GGT, NapA, OipA, HpaA, FlaA, FecA, BabA, and SabA were merged with the cholera toxin subunit B as an adjuvant [51]. These studies targeted the construction of vaccines for humans while some vaccines had been constructed for mice too using HP1453, HP0906 and HP0487 hypothetical antigens [52] and UreB, HpaA, and NapA proteins [49]. Although all these proteins are antigenic and were good candidate vaccines, but none of the prior efforts had tried to analyse the whole proteome with a structural vaccinology approach to identify the suitable antigens, especially the secretory/outer membrane antigens. They tried to identify the MHCII epitopes based on alleles, but the selection criteria for the alleles were not described. In contrast, we selected MHCII epitopes with a high affinity toward MHCII alleles whose expression was found in *H. pylori*-related gastric cancer, gastric ulcer, gastritis etc. [18–20, 22]. Our vaccine is predicted to cover 86.04% of the world's population and can raise a good humoral immune response. Therefore, the chance of success for our vaccine candidate is higher, compared to similar approaches tried earlier.

Finally, to prepare this construct ready for future downstream processing, we did in silico molecular cloning (Additional file 1: Fig. S4). To achieve higher expression, we used the *E. coli* expression system, which is an ideal choice for recombinant protein expression in large quantity [54]. The GC content (57.11%) and CAI score (1) were favourable for high expression while 6X His-tag was added to make it ready for straight forward purification of the expressed protein, if required.

## Conclusion

We have attempted to construct a multi-chimaera vaccine for *H. pylori* by choosing secretory/outer membrane protein as the target. The constructed vaccine comprises of epitopes capable of IFN-γ expression on T- cells, and B-cells epitopes which can generate a robust cell-mediated immunity as well as humoral response. This vaccine has the potential to be protective across diverse population groups across the globe. The constructed vaccine is also well characterised by different physicochemical properties as well as its antigenic and allergenic profiles. The docking study and immune simulation revealed a good binding with TLRs and cell-mediated and humoral immune responses, respectively. We also performed *in-silico* cloning to ensure the successful purification of the constructed vaccine for future use. Further, in-vitro validation is needed to guarantee the control of *H. pylori* infection*.* We expect this vaccine shows a promising result to control *H. pylori* infection.

## Methods

### Protein collection and ESP identification

*H. pylori* (Puno135) whole proteome was obtained from NCBI repository. To identify the excretory/outer membrane proteins, we employed the “SecretomeP 2.0 server” (“http://www.cbs.dtu.dk/services/SecretomeP/”) to identify the non-classical excreted proteins by selecting the gram-negative bacteria with a threshold of 0.6. Additionally, the “SignalP 6.0” (“http://www.cbs.dtu.dk/services/SignalP/”) was employed for standard Sec/SPI signal peptides [55]. To differentiate between signal peptides and similar N-terminal transmembrane segments, we utilized the DeepSig prediction server, which is based on a deep convolutional neural network (“https://deepsig.biocomp.unibo.it/welcome/default/index”) to distinguish between the signal peptide and similar N-terminal transmembrane segments [56]. The “LipoP server” (“http://www.cbs.dtu.dk/services/LipoP/”) was used to exclude cytoplasmic proteins. Default settings were used for all these analyses to increase the coverage [57]. The proteins predicted by these servers were merged, and the resulting set was scanned for the identification and localization of transmembrane helices using the “TMHMM 2.0” (“http://www.cbs.dtu.dk/services/TMHMM/”). Proteins predicted to have less than one transmembrane motif were considered secreted/outer membrane proteins. These results were further confirmed by the “Phobius” server, which identified proteins with a signal peptide but no transmembrane region [53].

### Identifying *H. pylori*-specific proteins

The proteins predicted in the earlier step were analyzed for allergenicity using the “AlgPred server” (“https://webs.iiitd.edu.in/raghava/algpred/”). This server provides results with 85% precision at a threshold of − 0.4, making it highly reliable. The non-allergic proteins were then evaluated for their antigenicity using the “ANTIGENpro” software (“http://scratch.proteomics.ics.uci.edu/”). Furthermore, the antigenic proteins were subjected to BLAST for homology findings with humans. Proteins having an identity score of ≥ 50% with a host protein were discarded to avoid cross-reactivity.

### Prediction of T_H_-cell, B-cell and IFN-γ-inducing epitopes

To identify MHC II binding epitopes, specific HLA alleles including HLA-DQA1*02*01, HLA-DQA1*01*02, DQB1*01*401, HLA-DQA1*01*03, HLA-DPA1*01*01 and HLA-DPB1*09*01 were selected based on their reported expression in *H. pylori*-related gastric cancer, gastric ulcer, gastritis etc. [18–20]. The 15-mer MCH II binding peptides were predicted by using”NetMHCIIpan-4.0” and “NetMHCII-2.3″ (“http://www.cbs.dtu.dk/services/NetMHCII/”) server (“http://www.cbs.dtu.dk/services/NetMHCIIpan/”) by keeping the strong binder threshold 3. Both of these servers employ Artificial Neural Networks to provide comprehensive information on MHC II epitopes (14–16). From the predicted MHC II binding epitopes, IFN-γ inducing epitopes were identified by the “IFNepitope server" (“http://crdd.osdd.net/raghava/ifnepitope/predict.php”) with a filter set to Motif hybrid and SVM approach. Epitopes with a score > 1 were chosen to be included in the vaccine. Proteins having common peptides in MHC II and IFN-γ epitope were selected for further analysis. To produce a long-lasting humoral immune response, the B-cell epitope plays a significant role. The “BCPred” (“http://ailab-projects1.ist.psu.edu:8080/bcpred/”) tool was utilized to design a 12mer B-cell epitope using a specificity threshold of 75%.

To confirm whether the constructed vaccine may provoke any allergic response, the “AlgPred” server was applied. Additionally, two servers, 1) “ANTIGENpro’ software (“http://scratch.proteomics.ics.uci.edu/”) and 2) ‘Vaxijen *v*2.0” (“http://www.ddg-pharmfac.net/vaxijen/VaxiJen/VaxiJen.html”) were utilised to predict the antigenic characteristics of the selected epitopes.

### Population coverage and toxicity prediction of selected vaccine candidate

Considering that *H. pylori* is a global concern, and to select the good epitopes for its vaccine construction, the human population coverage needed to be considered. To check the world population coverage of derived HTL epitopes, we utilized the “IEDB population coverage tool” (“http://tools.iedb.org/population/”). The prediction was made by using the World and Class II options and selecting the alleles for individual epitopes [58]. Furthermore, the toxicity prediction of the selected peptide, “ToxinPred” (“https://webs.iiitd.edu.in/raghava/toxinpred/index.html”) tool was utilised by using the SVM (Swiss-Prot) based method [59].

### Merging epitopes for multiepitope vaccine

The non-allergic HTL and B-cell epitopes were chosen to create the vaccine construct. The B-cell and HTL epitopes were merged by linker GPGPG. Additionally, β-defensin 2 (adjuvant) (PDB ID: 1FD3_A) was attached at the N-terminal using linker EAAAK to improve vaccine immunogenicity.

### Physiochemical properties estimations and secondary (2D) structure prediction

To ensure that the constructed vaccine candidate should have no allergenicity and a good antigenic score, so rechecked the allergenicity and antigenicity. To check the stability and physiochemical properties, we used the ProtParam tool (“https://web.expasy.org/protparam/”) which calculates the MW, half-life, composition of AA, GRAVY index, theoretical pI and instability index etc. of the vaccine construct. We used PSIPRED (“http://bioinf.cs.ucl.ac.uk/psipred/”) for the computation of the 2D structure of the designed vaccine. It uses PSI-Blast to predict β-sheet, α-helix, turns, and coils of the 2D structure based on the presence of homologous proteins to our vaccine. For the prediction, PSIPRED 4.0 (Predict secondary structure) option was selected.

### Prediction, refinement and estimation of tertiary (3D) structure

The “Raptor X server” (“http://raptorx.uchicago.edu/”), a widely used online web server based on Deep learning to predict the 3D structure of the constructed model, was employed which also estimates the RMSD value [60]. Pymol was used to visualise the 3D structure. For further improvement, we utilised “Galaxy refine 2” (“http://galaxy.seoklab.org/”) which uses the CASP10 technique for structure relaxation [61]. Subsequently, the “YASARA software for energy minimization” was used on the refined model obtained from Galaxy Refine [62]. For 3D structure validation we used “ProSA-web” (“https://prosa.services.came.sbg.ac.at/prosa.php”), “SAVES v6.0” (“https://saves.mbi.ucla.edu/”). The "ProSA" server evaluates and validates the structure's overall quality by utilizing the -Z score. SAVES 6.0 includes ERRAT, which assesses the overall quality factor, and "PROCHECK," which analyses the Ramachandran plot.

### Prediction of discontinuous B-cell epitope

The folded configuration of the developed vaccine possesses both conformational and discontinuous B-cell epitopes. According to the report, it is believed that over 90% of B-cell epitopes are of the discontinuous type [63]. To predict these discontinuous B-cell epitopes within the constructed vaccine, we utilized “the ElliPro server” (“http://tools.iedb.org/ellipro/”) by selecting the 0.5 threshold and 6 Å distance. [64].

### Proteasomal processing

To check for the presence of chimeric class I MHC epitopes, we used the "Proteasomal Cleavage Prediction tool" of IEDB (“http://tools.iedb.org/netchop/help/”) using the NetChop option and the "Proteasome Cleavage Prediction Server of Immunomedicine group" (“http://imed.med.ucm.es/Tools/pcps/"). For both the servers 0.5 threshold option was selected [65, 66].

### Molecular docking

To trigger any immune response, the receptors play a very crucial role. In *H. pylori* infection involvement of TLRs has been widely explored [40, 41, 67]. For docking the vaccine with TLRs, we retrieved the PDB structure of TLR 2, 4 and 5 for which PDB IDs were 6NIG, 4G8A and 3J0A, respectively from the PDB database (“https://www.rcsb.org/”) and did the blind docking using the “ClusPro 2.0” tool (“https://cluspro.org/help.php”). This server provides 10 docked models along with their RMSD and binding energy scores, which reflect the efficiency of the docking process. Using the information obtained from ClusPro, we further performed docking of the receptors and ligands using the "HADDOCK 2.4" server. This server requires the interacting residues, which were predicted based on the results from ClusPro. HADDOCK provides output in terms of RMSD, desolvation energy, Z score, and Van der Waals forces, which provide insights into the docking interactions [68]. The resulting docked models were visualized using Pymol software, and interaction mapping was performed using the "LigPlot + v.2.2" program [69].

### MD simulation

For molecular dynamics (MD) simulation, the PBD file of the construct, TLR-2, 4 and 5 and docked complex of Vacc-TLR-2, 4 and 5 were used. The MD simulation was performed using GROMACS (GROningen MAchine for Chemical Simulations) 2020.5 version. During the simulation, the Ff99SB force field was employed, and the system was immersed in a cubic box utilizing the TIP3P water model. Additionally, the neutralization process involved the addition of Na or Cl ions. After the energy minimisation, solvent equilibration was performed through temperature (NVT) and pressure (NPT) run for 1 ns. The MD simulation was performed for 100 ns to produce trajectory files.

### Immune simulation

To characterise the immune profile of the constructed multiepitope vaccine, we used the “C-ImmSimm server” (“https://kraken.iac.rm.cnr.it/C-IMMSIM/”). This server utilizes a position-specific scoring matrix machine learning method to predict immune response and immune epitope interactions. For simulation purposes, three injection volumes of 10 µl at the time interval of 0, 84 and 168 days with a simulation step of 1000 were chosen [70].

### In-silico cloning

The reverse translated AA sequence of the vaccine was optimized for expression in *Escherichia coli* (strainK12) bacterium using “Java Codon Adaptation Tool” (JCat) (“http://www.prodoric.de/JCat”) [71]. The selection of NdeI and BmtI restriction sites was made for the purpose of incorporating the sequence. Subsequently, the SnapGene tool was employed to insert the sequence into the pET-28b (+) vector.

### Supplementary Information

**Additional file 1**. Supplementary figures and tables.

## Data Availability

All relevant data used in this study are included in the manuscript and supplementary files.

## Abbreviations

WHO: World Health Organisation
PRRs: Pattern recognition receptors
PAMPs: Pathogen-associated molecular patterns
GC: Gastric cancer
DALYs: Disability Adjusted Life Years
TLRs: Toll-like receptors
ESP: Excretory secretory product
JCat: Codon adaptation tool
CAI: Codon adaptation index
2D: 2 Dimension structure
3D: 3 Dimension structure
pI: Theoretical isoelectric point
GRAVY: Grand average of hydropathicity
PDB: Protein Data Bank
IL-2: Interleukin-2
IFN-γ: Interferon-gamma
MHC-II: Major histocompatibility complex-II
IEDB: Immune Epitope Database
NCBI: National Centre for Biotechnological Information
MW: Molecular weight
MD: Molecular dynamics
