# Supplementary material for: Designing and development of multi-epitope chimeric vaccine against Helicobacter pylori by exploring its entire immunogenic epitopes: an immunoinformatic approach
Source: BMC Bioinformatics. 2023 Sep 22;24:358. doi: 10.1186/s12859-023-05454-2 (PMC10517479; doi:10.1186/s12859-023-05454-2)
Supplement: Supplementary file 1 — Additional file 1. Supplementary figures and tables. [file 12859_2023_5454_MOESM1_ESM.docx]

**Designing and development of multi-epitope chimeric vaccine against *Helicobacter pylori* by exploring its entire immunogenic epitopes: an immunoinformatic approach**

Anand K Keshri^1^, Rimanpreet Kaur^1^, Suraj S Rawat^1^, Naina Arora^1^, Rajan K Pandey^2^, Bajarang V Kumbhar^3^, Amit Mishra^4^, Shweta Tripathi^1^*, Amit Prasad^1^*

**Supplementary Material Caption:**

**Fig S1**: (A) The overall energy plot from the ProSA server of the constructed vaccine shows low energy levels, indicating its stability and energetically favoured and likely to adopt a stable conformation. (B) The 3D-1D ratio of the vaccine structure is determined by the Verify3D tool. The average score of 0.2 throughout the structure suggests a good agreement between the experimental structure and the expected features based on the amino acid sequence. (C) the overall quality factor of the constructed vaccine, as assessed by the ERRAT tool. A value of 77.455 indicates high overall quality, indicating that the vaccine structure is reliable and has a high degree of accuracy.

**Fig S2**: Molecular interaction studies were conducted to analyze the interactions of the vaccine construct with TLRs, (A-C) TLR2, TLR4, and TLR5, respectively; (i) HADDOCK score versus an interface-RMSD measure of the quality of the predicted protein-protein complex, with lower scores indicating better complexes. (ii) Electrostatic energy of interacted complex versus interface-RMSD. The electrostatic energy reflects the strength of the electrostatic interactions between the vaccine construct and the TLRs. (iii) HADDOCK score versus the fraction of frequent contacts; The HADDOCK score represents the overall quality of the predicted complex, while the fraction of frequent contacts measure the percentage of contacts that occur frequently during molecular dynamics simulations. (iv) Vander Waals energy versus interface-RMSD (FCC); where FCC refers to the Functional Contact Calculation. Vander Waals energy represents the strength of the Vander Waals interactions between the vaccine construct and the TLRs. (v) and (vi) show the Vander Waals energy and electrostatic energy, respectively, of each cluster within the predicted complexes. Clusters are subsets of the predicted complexes with distinct conformations.

**Fig S3:** The figure representing the C-ImmSim presentation of an in silico immune simulation with the constructed vaccine. (A) Diploid cell population (B) Regulatory T cell population (C) Cytotoxic T cell population (D) Natural Killer cell population (E) Dendritic cell population (F) Eosinophil cell population.

**Fig S4:** *In-silico* restriction cloning of the final vaccine was performed in the pET28b (+) vector, where the red color represents the portion of the vaccine inserted between the NdeI and BmtI restriction sites, and the black color represents the vector backbone.

**Table S1:** List of all selected 25 proteins with their score from Secretome, SignalP, DeepSig, LipoP, TMHMM and Phobius servers.

**Table S2:** Scores obtained from the molecular interaction analysis of TLRs with the vaccine were determined using the Haddock server.

**Table S3:** Toxicity evaluation of the selected peptide used for vaccine construction was performed based on SVM (Swiss-Prot) along with the SVM score, hydrophobicity, hydropathicity, hydrophilicity, and charge present on the individual peptide.

**
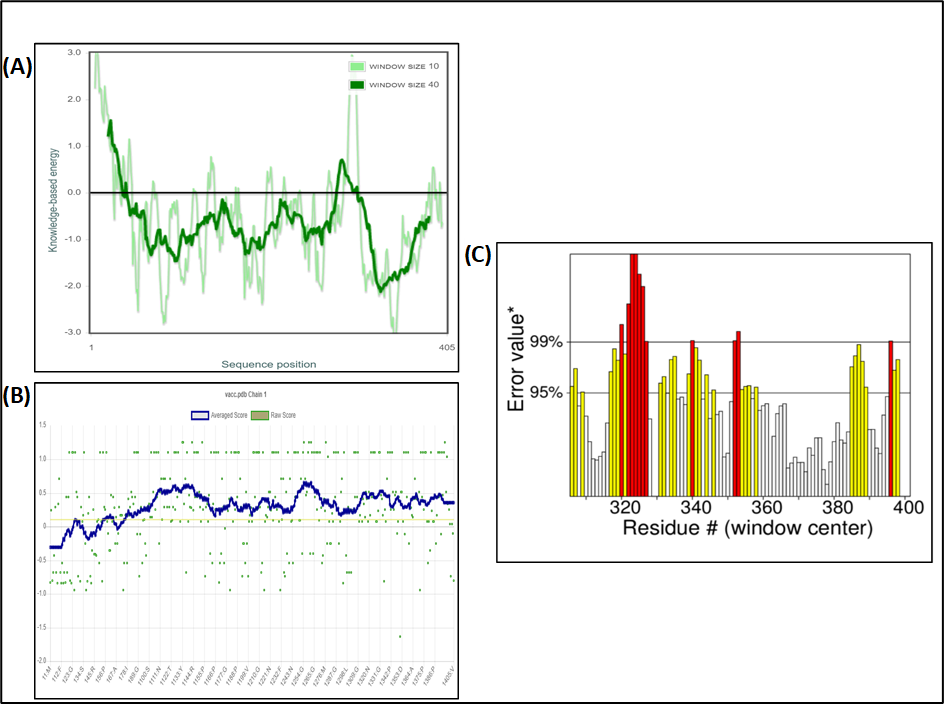
**

**Fig S1**: (A) The overall energy plot from the ProSA server of the constructed vaccine shows low energy levels, indicating its stability and energetically favoured and likely to adopt a stable conformation. (B) The 3D-1D ratio of the vaccine structure is determined by the Verify3D tool. The average score of 0.2 throughout the structure suggests a good agreement between the experimental structure and the expected features based on the amino acid sequence. (C) the overall quality factor of the constructed vaccine, as assessed by the ERRAT tool. A value of 77.455 indicates high overall quality, indicating that the vaccine structure is reliable and has a high degree of accuracy.


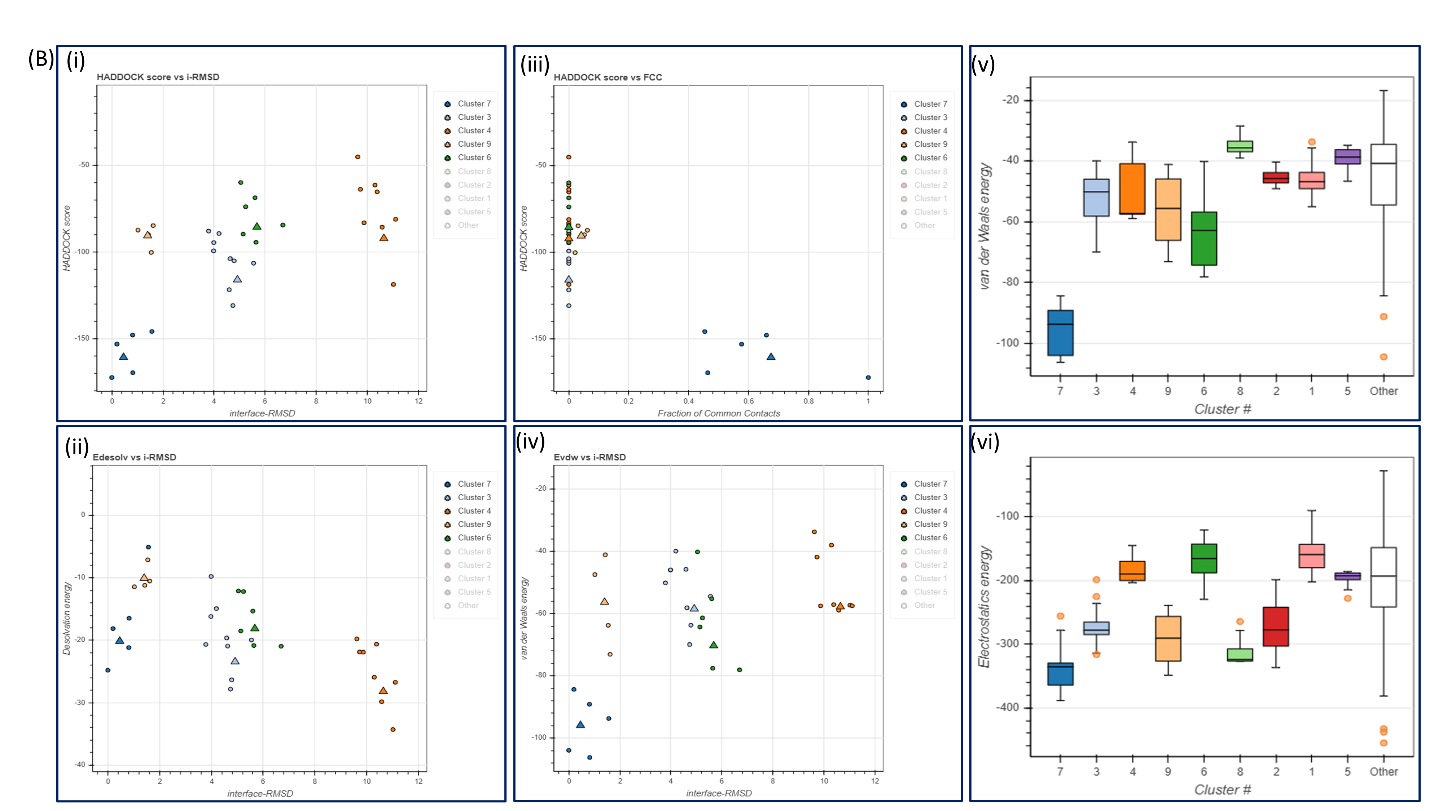
**
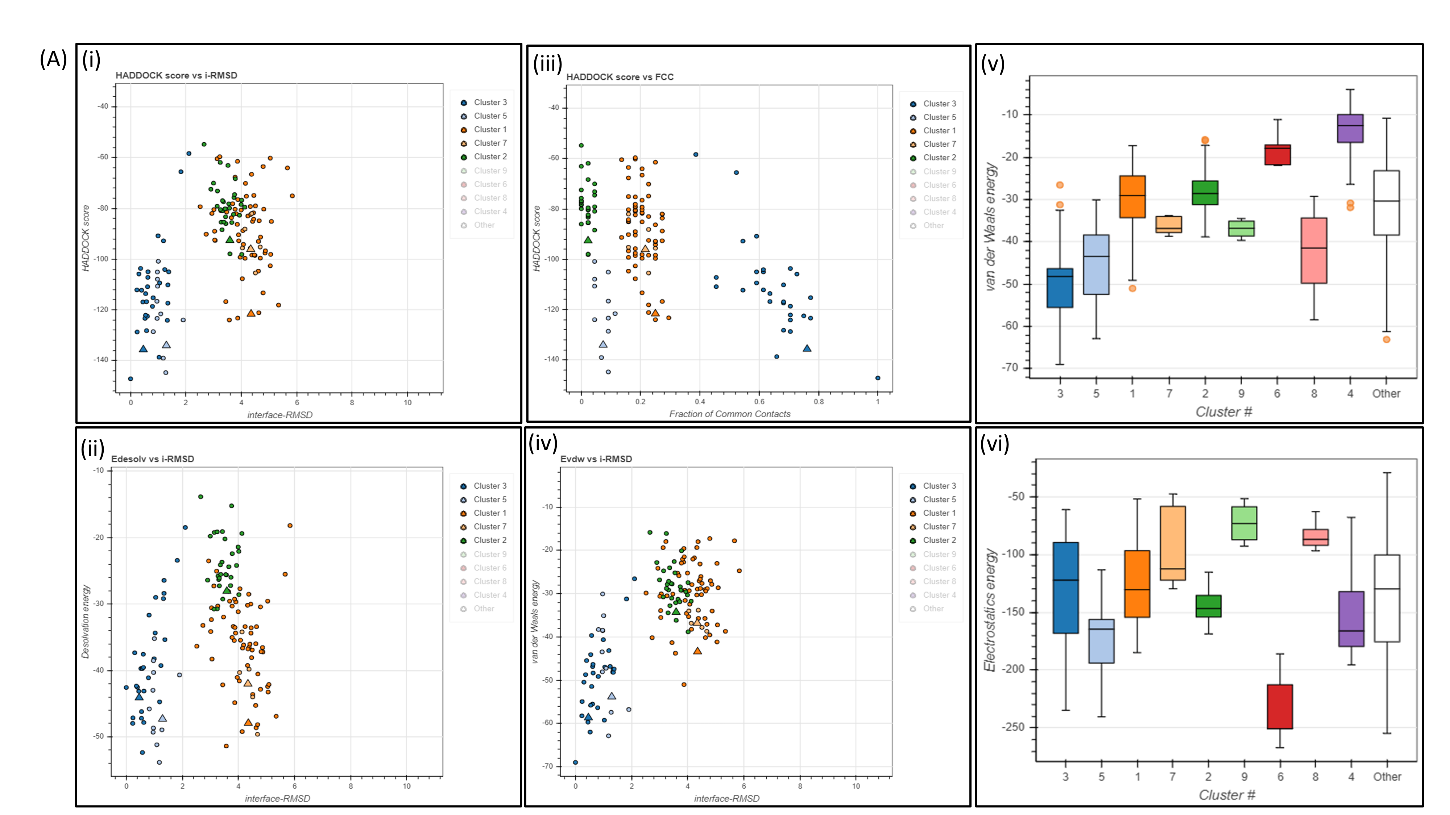
**


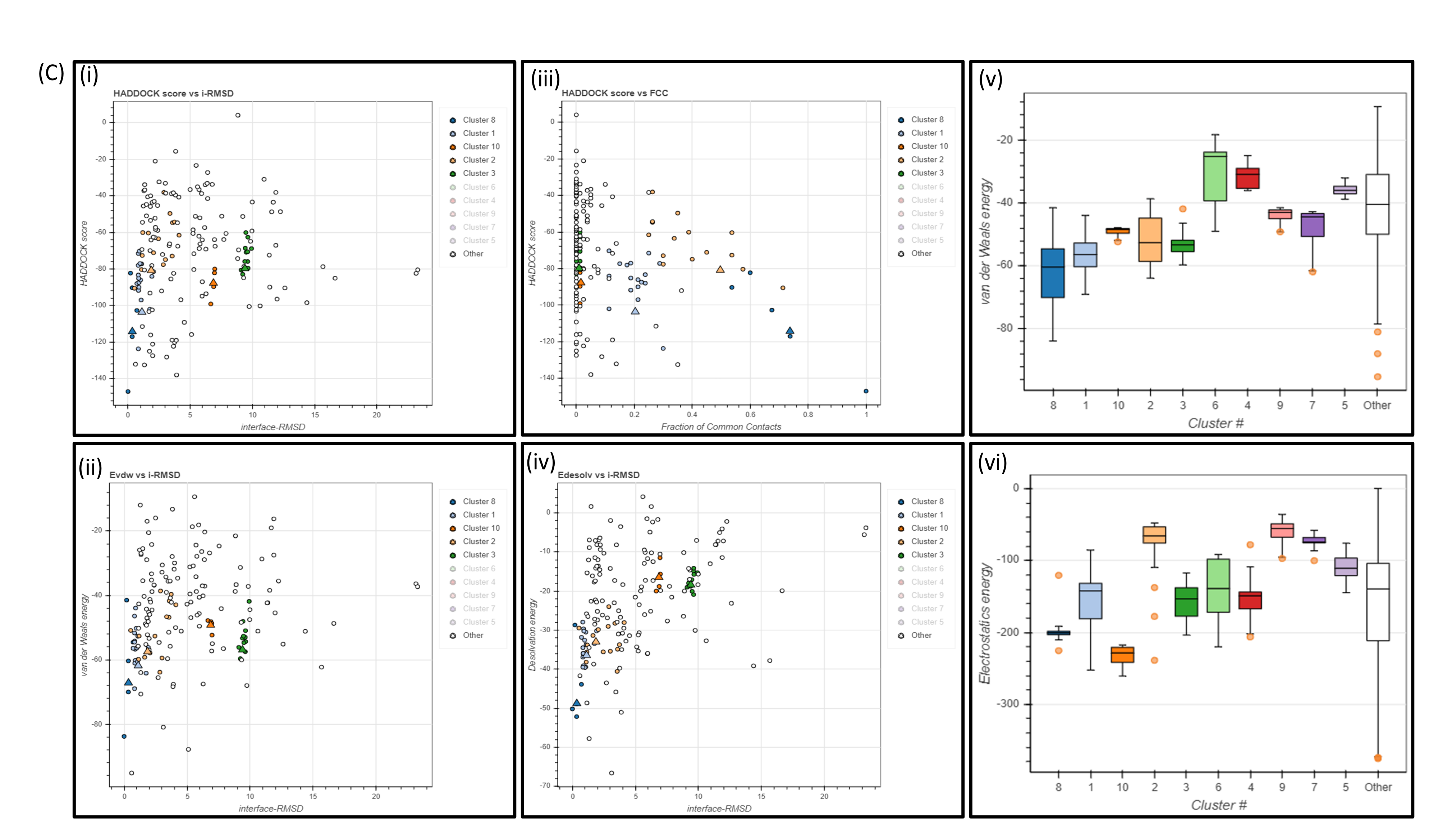


**Fig S2**: Molecular interaction studies were conducted to analyze the interactions of the vaccine construct with TLRs, (A-C) TLR2, TLR4, and TLR5, respectively; (i) HADDOCK score versus an interface-RMSD measure of the quality of the predicted protein-protein complex, with lower scores indicating better complexes. (ii) Electrostatic energy of interacted complex versus interface-RMSD. The electrostatic energy reflects the strength of the electrostatic interactions between the vaccine construct and the TLRs. (iii) HADDOCK score versus the fraction of frequent contacts; The HADDOCK score represents the overall quality of the predicted complex, while the fraction of frequent contacts measure the percentage of contacts that occur frequently during molecular dynamics simulations. (iv) Vander Waals energy versus interface-RMSD (FCC); where FCC refers to the Functional Contact Calculation. Vander Waals energy represents the strength of the Vander Waals interactions between the vaccine construct and the TLRs. (v) and (vi) show the Vander Waals energy and electrostatic energy, respectively, of each cluster within the predicted complexes. Clusters are subsets of the predicted complexes with distinct conformations.


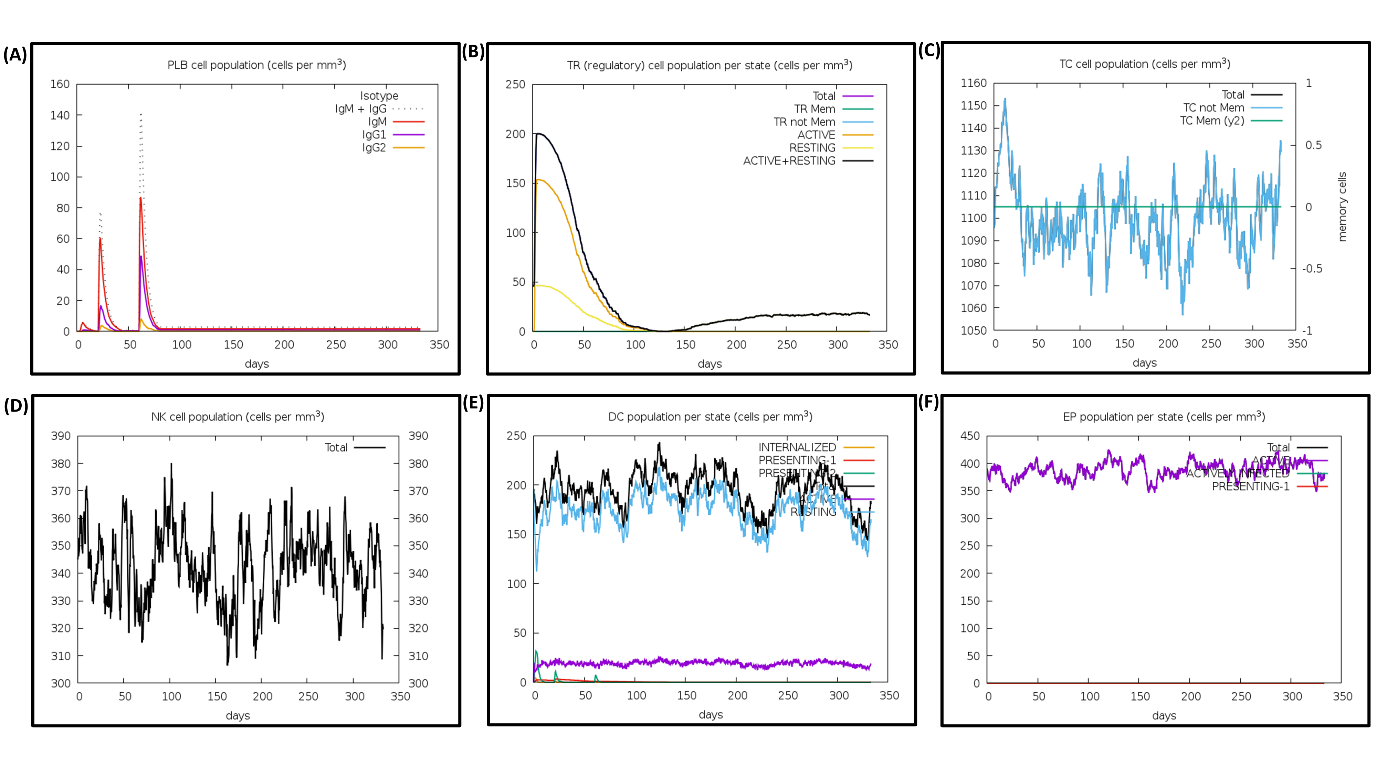


**Fig S3:** The figure representing the C-ImmSim presentation of an in silico immune simulation with the constructed vaccine. (A) Diploid cell population (B) Regulatory T cell population (C) Cytotoxic T cell population (D) Natural Killer cell population (E) Dendritic cell population (F) Eosinophil cell population.

**
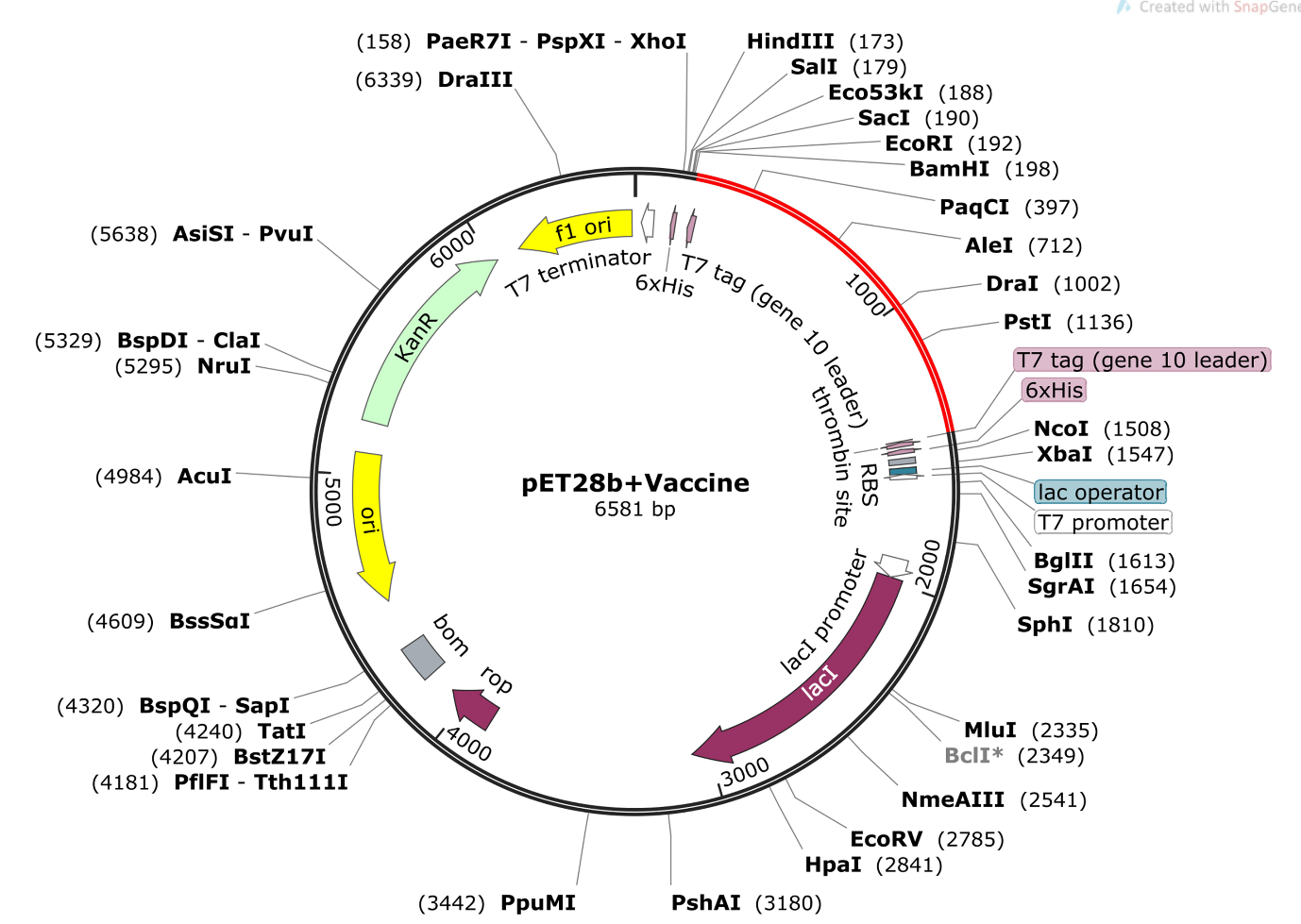
**

**Fig S4:** *In-silico* restriction cloning of the final vaccine was performed in the pET28b (+) vector, where the red color represents the portion of the vaccine inserted between the NdeI and BmtI restriction sites, and the black color represents the vector backbone.

| **Accession id** | ***H. pylori* Protein** | **Secretome** | **SignalP** | **DeepSig** | **LipoP** | **TMHMM** | **Phobius** |
| --- | --- | --- | --- | --- | --- | --- | --- |
| WP_000395384.1 | Hop family outer membrane protein HopE | 0.87the 752 | 0.99755 | 0.88 | 18.7461 | 0 | 0 |
| WP_000592746.1 | 5'-nucleotidase, lipoprotein e(P4) family | 0.58267 | 0.9733 | 0.88 | 11.333 | 0 | 0 |
| WP_000751487.1 | Hop family adhesin HopQ | 0.95651 | 0.9968 | 0.86 | 18.7385 | 0 | 0 |
| WP_000715036.1 | Outer membrane protein | 0.93145 | 0.99175 | 0.88 | 12.8702 | 0 | 0 |
| WP_000716258.1 | Hop family adhesin BabA | 0.95598 | 0.98618 | 0.83 | 12.9136 | 0 | 0 |
| WP_000720369.1 | LPP20 family lipoprotein | 0.83914 | 0.98195 | 0.88 | 14.1907 | 0 | 0 |
| WP_000591195.1 | Hop family outer membrane protein HopL | 0.95651 | 0.6422 | 0.76 | 13.8246 | 0 | 0 |
| WP_000731571.1 | Copper resistance determinant CrdA | 0.88813 | 0.98294 | 0.88 | 12.7363 | 0 | 0 |
| WP_000738924.1 | YceI family protein | 0.65515 | 0.87642 | 0.88 | 9.42819 | 0 | 0 |
| WP_001000224.1 | Flagellar hook protein FlgE | 0.92458 | 0.7312 | 0.72 | 11.7321 | 0 | 0 |
| WP_000750187.1 | Outer membrane protein | 0.92555 | 0.94385 | 0.88 | 12.8375 | 0 | 0 |
| WP_000750999.1 | S-adenosyl-l-methionine hydroxide adenosyltransferase family protein | 0.7773 | 0.50708 | 0.87 | 11.9174 | 0 | 0 |
| WP_000885486.1 | Flagellin A | 0.95258 | 0.7423 | 0.78 | 13.2514 | 0 | 0 |
| WP_000754047.1 | TolC family protein | 0.57714 | 0.90559 | 0.88 | 9.81222 | 0 | 0 |
| WP_000788620.1 | Hop family outer membrane protein HopF | 0.771 | 0.76343 | 0.83 | 10.6228 | 0 | 0 |
| WP_000819957.1 | Alginate lyase family protein | 0.52614 | 0.99425 | 0.88 | 14.0948 | 0 | 0 |
| WP_000971468.1 | Outer membrane protein | 0.9387 | 0.5469 | 0.73 | 13.2156 | 0 | 0 |
| WP_000976512.1 | Hop family adhesin AlpA | 0.91344 | 0.9982 | 0.88 | 19.3578 | 0 | 0 |
| WP_000521812.1 | Septal ring lytic transglycosylase RlpA family protein | 0.92223 | 0.9981 | 0.75 | 11.231 | 0 | 0 |
| WP_000726394.1 | Outer membrane beta-barrel protein HofC | 0.95055 | 0.96659 | 0.86 | 13.5689 | 0 | 0 |
| WP_000751487.1 | Hop family adhesin HopQ | 0.95814 | 0.99678 | 0.88 | 18.7385 | 0 | 0 |
| WP_000914869.1 | sel1 repeat family protein | 0.9292 | 0.51004 | 0.88 | 14.5191 | 0 | 0 |
| WP_000682011.1 | Hypothetical protein | 0.95115 | 0.95181 | 0.88 | 18.2747 | 0 | 0 |
| WP_000739478.1 | Peptidylprolyl isomerase CBF2 | 0.62751 | 0.95512 | 0.88 | 15.2405 | 0 | 0 |
| WP_080004620.1 | Outer membrane beta-barrel protein | 0.94687 | 0.99334 | 0.78 | 11.6034 | 0 | 0 |

**Table S1:** List of all selected 25 proteins with their score from Secretome, SignalP, DeepSig, LipoP, TMHMM and Phobius servers.

|  | **TLR-2 docked complex** | **TLR-4 docked complex** | **TLR-5 docked complex** |
| --- | --- | --- | --- |
| **HADDOCK score** | -135.7 +/- 7.8 | -160.8 +/- 10.5 | -114.3 +/- 21.1 |
| **Cluster size** | 29 | 5 | 5 |
| **RMSD from the overall lowest-energy structure** | 1.5 +/- 1.0 | 1.5 +/- 1.3 | 1.7 +/- 1.0 |
| **Van der Waals energy** | -58.7 +/- 6.6 | -95.9 +/- 9.4 | -67.2 +/- 11.1 |
| **Electrostatic energy** | -179.7 +/- 35.1 | -336.1 +/- 49.9 | -186.8 +/- 39.7 |
| **Desolvation energy** | -44.1 +/- 6.6 | -20.1 +/- 3.2 | -48.8 +/- 3.1 |
| **Restraints violation energy** | 30.7 +/- 14.0 | 224.1 +/- 110.8 | 390.8 +/- 51.4 |
| **Buried Surface Area** | 1950.4 +/- 143.0 | 3257.9 +/- 325.1 | 2368.3 +/- 245.6 |
| **Z-Score** | -1.6 | -2.4 | -1.8 |

**Table S2:** Scores obtained from the molecular interaction analysis of TLRs with the vaccine were determined using the Haddock server.

| **Peptide ID** | **Peptide Sequence** | **SVM Score** | **Prediction** | **Hydrophobicity** | **Hydropathicity** | **Hydrophilicity** | **Charge** |
| --- | --- | --- | --- | --- | --- | --- | --- |
| Seq1 | KHESLRAYENAKDYD | -0.39 | Non-Toxin | -0.46 | -1.93 | 0.91 | -0.50 |
| Seq2 | GEIYNMYAHTAAHKT | -1.42 | Non-Toxin | -0.11 | -0.66 | -0.32 | 1.00 |
| Seq3 | ISNVYSAKVNTANFQ | -1.53 | Non-Toxin | -0.10 | -0.15 | -0.44 | 1.00 |
| Seq4 | ARPKKKDSHHAAQHG | -1.20 | Non-Toxin | -0.47 | -2.01 | 0.83 | 4.50 |
| Seq5 | NEPIDAITNRKLNIS | -1.42 | Non-Toxin | -0.24 | -0.66 | 0.32 | 0.00 |
| Seq6 | SNNIANVNTLGYRSN | -0.89 | Non-Toxin | -0.23 | -0.78 | -0.25 | 1.00 |
| Seq7 | NSNQTGVRAHASVIT | -0.88 | Non-Toxin | -0.17 | -0.34 | -0.19 | 1.50 |
| Seq8 | SGANYNAVIASGNQN | -0.92 | Non-Toxin | -0.09 | -0.47 | -0.37 | 0.00 |
| Seq9 | HTNFSNSRAANAISP | -0.72 | Non-Toxin | -0.20 | -0.68 | -0.15 | 1.50 |
| Seq10 | ATAGFFVGVNFAGNT | -1.54 | Non-Toxin | 0.17 | 0.84 | -0.83 | 0.00 |
| Seq11 | QAYLRSAGADVSYRR | -1.30 | Non-Toxin | -0.35 | -0.78 | 0.23 | 2.00 |
| Seq12 | ARNQVQNAQNQANNY | -0.91 | Non-Toxin | -0.43 | -1.85 | -0.03 | 1.00 |
| Seq13 | TSNTNSANNTNS | -0.67 | Non-Toxin | -0.36 | -1.68 | 0.02 | 0 |
| Seq14 | TTDGGKNSCQTF | -0.18 | Non-Toxin | -0.25 | -1.07 | 0.17 | 0 |
| Seq15 | DPKNKTPINDT | -0.55 | Non-Toxin | -0.43 | -1.99 | 0.89 | 0 |
| Seq16 | AEESANFNKNNI | -0.77 | Non-Toxin | -0.28 | -1.23 | 0.4 | -1 |
| Seq17 | GTITCGDTTPAS | -0.77 | Non-Toxin | -0.04 | -0.06 | -0.13 | -1 |
| Seq18 | KGYNHSQEVEKV | -0.92 | Non-Toxin | -0.35 | -1.59 | 0.58 | 0.5 |

**Table S3:** Toxicity evaluation of the selected peptide used for vaccine construction was performed based on SVM (Swiss-Prot) along with the SVM score, hydrophobicity, hydropathicity, hydrophilicity, and charge present on the individual peptide.
